# Response to ‘Upgrade procedures of pacemakers or implantable cardioverter-defibrillators require creative clinical decision making and careful planning that begins at the first implantation’

**DOI:** 10.1093/europace/euag240

**Published:** 2026-09-25

**Authors:** Daniel Keene, Nandita Kaza, Haran Burri, Archana Rao, Jens Cosedis Nielsen

**Affiliations:** National Heart and Lung Institute, Imperial College London, London, UK; National Heart and Lung Institute, Imperial College London, London, UK; Department of Cardiology, University Hospital of Geneva, Geneva, Switzerland; Department of Cardiology, Liverpool Heart and Chest Hospital, Liverpool, UK; Department of Cardiology, Aarhus University, Aarhus, Denmark

**Response to Letter to the Editor refers to ‘Upgrade procedures of pacemakers or implantable cardioverter-defibrillators require creative clinical decision making and careful planning that begins at the first implantation’ by Alexander H. Maass *et al*.,** https://doi.org/10.1093/eurospace/euag174.

We thank Maass *et al.* for their thoughtful comments on our consensus statement on device upgrades and downgrades^1^ and welcome the opportunity to clarify several points.

We agree that many of the principles underpinning decision-making for device upgrades are equally applicable to the management of lead dysfunction. In both settings, the balance between lead extraction, lead abandonment and implantation of additional hardware should be tailored, taking into account patient characteristics, venous access, infection risk and future treatment requirements.

With regard to lead selection, we recognize that both DF-1 and DF-4 systems have advantages and disadvantages.^2^ While future flexibility is an important consideration, it represents only one of many factors influencing lead choice. Decisions at the time of initial ICD implantation should primarily reflect the patient's contemporary clinical needs and the available evidence, rather than a future scenario that may never arise. We therefore do not believe that current evidence supports the routine use of DF-1 leads in all patients undergoing ICD implantation in anticipation of possible future downgrading. We acknowledge the increasing recognition that, although DF-4 systems have simplified ICD implantation and become the contemporary standard, this has come at the expense of reduced flexibility in selected clinical scenarios. Although DF-1 systems may more readily facilitate selected downgrade strategies, integrated DF-4 leads simplify implantation by reducing header connections and opportunities for connection errors, reduce header and generator bulk, and have become the contemporary standard with an established safety and reliability profile. Neither system is universally superior; rather, each offers distinct advantages depending on the clinical scenario, and lead selection should therefore remain individualized rather than driven by hypothetical future requirements.

We agree that, in many patients undergoing ICD deactivation, replacement with a like-for-like ICD generator and deactivation of tachyarrhythmia therapies represents the simplest—and often safest—strategy. In the elderly and frail population in whom this is typically considered, implanting an additional pacing lead or replacing a functioning DF-4 lead may expose patients to disproportionate procedural risk.

Regarding venous access, we agree that contralateral lead implantation and tunnelling should generally be avoided where possible, given the cumulative lead burden within the superior vena cava and the potential implications for future venous access and device infection. We also acknowledge the authors’ experience with brachial venoplasty and agree that it represents a valuable technique in experienced hands. Although published series report excellent procedural success, these results derive largely from experienced centres and may not be readily generalizable to all operators or institutions.^3^ However, whether establishing venous access before opening the device pocket confers a net procedural advantage during upgrade procedures remains uncertain, as existing leads may still be vulnerable to inadvertent damage during venous instrumentation and the risk of introducing infection is not eliminated even if the device pocket remains unopened. Furthermore, despite successful venoplasty, definitive axillary or subclavian access is still required and elastic recoil may occasionally limit subsequent venous puncture. We therefore believe that the optimal strategy should continue to be guided by patient anatomy, operator expertise and procedural objectives.

We thank the authors for their valuable comments, which reinforce the central principle of the consensus statement that management of patients undergoing device revision should remain individualized.

## Data Availability

No new data were generated or analysed in support of this research.
